# *MTHFR* C677T rs1801133 and *TP53* Pro72Arg rs1042522 gene variants in South African Indian and Caucasian psoriatic arthritis patients

**DOI:** 10.1590/1678-4685-GMB-2023-0325

**Published:** 2025-01-10

**Authors:** Pragalathan Naidoo, Ajesh B. Maharaj, Terisha Ghazi, Anil A. Chuturgoon

**Affiliations:** 1 University of KwaZulu-Natal, Howard College, College of Health Sciences, School of Laboratory Medicine and Medical Sciences, Department of Medical Biochemistry, Durban, South Africa.; 2 Walter Sisulu University, Faculty of Health Sciences, Department of Internal Medicine and Therapeutics, Eastern Cape, South Africa.

**Keywords:** *MTHFR* C677T rs1801133, *TP53* Pro72Arg rs1042522, psoriatic arthritis, C-reactive protein levels, methotrexate treatment

## Abstract

*Methylenetetrahydrofolate reductase* (*MTHFR*) gene is involved in homocysteine and folic acid metabolism. Tumour suppressor protein *TP53* gene maintains cellular and genetic integrity. To date, no studies associated the *MTHFR* C677T rs1801133 and *TP53* Pro72Arg rs1042522 with CRP levels and methotrexate (a folic acid antagonist) treatment outcomes in psoriatic arthritis (PsA) patients. The present study aimed to investigate whether the *MTHFR* rs1801133 and *TP53* rs1042522 gene variants influences CRP levels and methotrexate treatment outcomes in South African Indian and Caucasian PsA patients. PsA patients (n=114) and healthy controls (n=100) were genotyped for the rs1801133 and rs1042522 using RFLP-PCR. (i) Results for rs1801133 genotyping: Caucasian patients had a higher frequency of the variant T-allele versus healthy Caucasian controls (40% versus 22%; OR=2.31, 95% CI=1.10-4.88, p=0.0379). Patients with the variant CT+TT genotypes had higher median CRP levels at baseline versus wildtype CC genotypes (11.70 (5.3-28.80) mg/mL versus 7.40 (5.00-15.05) mg/mL, p=0.0355). After 6 months of methotrexate treatment median CRP levels between genotypes reduced and remained similar. (ii) Results for rs1042522 genotyping: Indian patients had a higher frequency of the variant Arg-allele versus healthy Indian controls (42% versus 29%; OR=1.75, 95% CI=1.07-2.86, p=0.0275). In conclusion, patients with the *MTHFR* rs1801133 variant T-allele have elevated CRP levels, which can be ameliorated with methotrexate.

## Introduction

Psoriatic arthritis (PsA) is a heterogeneous, chronic inflammatory musculoskeletal disease associated with psoriasis, a chronic autoimmune skin disease affecting 2-3% of the world’s population and characterized by hyper-proliferative keratinocytes and scaly erythematous plaques, and further triggered by several immunological, genetic, and environmental factors (Sankowski *et al.,* 2013). Around 23.8% (20.1% - 27.6%) of patients with psoriasis develop PsA, with incidence rate ranges of 0.27 to 2.7 per 100 person-years (Alinaghi *et al.,* 2019). 

Disease-modifying antirheumatic drugs (DMARDs) commonly used by PsA patients includes methotrexate (folic acid antagonist; initial DMARD), sulfasalazine (anti-inflammatory; mild disease), leflunomide (pyrimidine synthesis inhibitor; alternative to methotrexate), and biologics such as etanercept, infliximab and adalimumab (TNF-α antagonists; administered if failed prior DMARD) (Salt and Frazier, 2010).

Folate, and to a smaller extent vitamin B2, B6 and B12, play a crucial role in regulating plasma homocysteine levels. Elevated homocysteine levels (hyperhomocysteinaemia) are indicative of folate deficiency (Muskiet, 2005, Ramkaran *et al*., 2015). Chronic immune-mediated disorders such as PsA and chronic plaque psoriasis are common in patients with hyperhomocysteinaemia (Kural *et al.,* 2003, Malerba *et al*., 2006). Methotrexate, a folic acid antagonist, is the most common drug used for the treatment of moderate to severe psoriasis and PsA, and it is the anchor drug in most treatment regimens (Auepemkiate, 2016).

Methylenetetrahydrofolate reductase (MTHFR), a rate-limiting enzyme involved in the metabolism of homocysteine and folic acid, catalyzes the reduction of 5′,10′-methylenetetrahydrofolate to 5′- methyltetrahydrofolate (5′-methyl-THF). The 5′-methyl-THF is considered the most abundant form of folate in the plasma and serves as a methyl donor, which is essential for converting homocysteine to methionine (Engbersen *et al.,* 1995, Liew and Gupta, 2015). 

The *MTHFR* gene is positioned on chromosome 1 at 1p36.3 with a length of 2.2kb (Ramkaran *et al.,* 2015). The most common single nucleotide polymorphism (SNP) of the *MTHFR* gene occurs at position 677 (rs1801133). It arises when a cytosine (C) residue of the *MTHFR* gene is converted to thymine (T), resulting in the substitution of alanine to a valine (Anderson *et al.,* 1997). This SNP negatively affects the thermostability of the MTHFR enzyme, resulting in hindered enzymatic activity at 37ºC or higher. The rs1801133 is therefore characterized as being “thermolabile” (Liew and Gupta, 2015, Ramkaran *et al.,* 2015). In addition, several studies have linked the rs1801133 variant T-allele with aberrantly increased plasma homocysteine levels in inflammatory disorders (Anderson *et al.,* 1997, Sun *et al.,* 2004, Brezovska-Kavrakova *et al.,* 2013, Nienaber-Rousseau *et al.,* 2013). Although the rs1801133 has been associated with various inflammatory disorders, limited and conflicting data is available on its role in psoriasis and PsA pathogenesis (Weger *et al.,* 2008, Liew *et al.,* 2012, Liew and Gupta, 2015).

The tumour suppressor protein, p53 is a transcription factor often referred to as the “guardian of the genome”. It plays an invaluable role in maintaining cellular and genetic stability and integrity in response to oxidative stress, DNA damage, cell cycle abnormalities, hypoxia and nutrient deficiencies (Barlev *et al.,* 2001, Vousden and Prives, 2009). P53 also plays a fundamental role in regulating key metabolic pathways, (Webster *et al.,* 1996, Kawauchi *et al.,* 2008, Assaily *et al.,* 2011) and immune response pathways (Levine, 2020). In addition, elevated p53 expression is associated with the pathogenesis of inflammatory psoriasis (Baran *et al.,* 2005, Moorchung *et al.,* 2015) and PsA (Salvador *et al.,* 2005, Assmann *et al.,* 2010).

The *TP53* gene is positioned on chromosome 17 at 17p13.1 with a length of 19kb (Vousden and Prives, 2009). The p53 proline (Pro)-72-arginine (Arg) (Pro72Arg) SNP (rs1042522), located at codon 72 on exon 4 of the *TP53* gene, alters the overall functionality of p53 leading to aberrant changes in DNA repair processes, cell cycle progression, apoptosis, and cellular senescence (Dumont *et al.,* 2003, Pim and Banks, 2004, Siddique and Sabapathy, 2006). The rs1042522 plays a role in the pathogenesis of several diseases, including diabetes (Bonfigli *et al.,* 2013), cancer (Proestling *et al.,* 2012), cardiovascular disease (Khan *et al.,* 2016) and rheumatoid arthritis (Lee *et al.,* 2012). Although the rs1042522 has been associated with various inflammatory disorders, limited and conflicting data is available on its role in psoriasis and PsA pathogenesis (Butt et al., 2006, Assmann *et al.,* 2010).

Within the context of a South African population, psoriasis and PsA are prevalent in the Caucasian and Indian populations but not in the indigenous African population (Maharaj and Tak, 2015). The aim of the present study was to investigate whether the *MTHFR* rs1801133 and *TP53* rs1042522 gene variants influences CRP levels and methotrexate treatment outcomes in South African Indian and Caucasian PsA patients, and to identify risk factors associated with PsA progression.

## Subjects and Methods

### Patient recruitment and sample collection

This study was approved by the Pharma-Ethics Research Ethics Committee of South Africa (Ethics reference number: 13095660). A detailed account on: (i) the recruitment of PsA patients (n = 114) and healthy controls (n = 100) for this study, (ii) blood collection from all study participants, (iii) biochemical testing [C-reactive protein (CRP) (biomarker of inflammation), total cholesterol, high-density lipoprotein (HDL) cholesterol, low-density lipoprotein (LDL) cholesterol, fasting plasma glucose, blood glycated haemoglobin (HbA1c), 25-hydroxy vitamin D [25(OH)D] and rheumatoid factor-immunoglobulin M (RF-IgM)], (iv) Health Assessment Questionnaire (HAQ) data collection, (v) demographical, anthropometric measurements, patient history and DMARDS data collection, and (vi) the inclusion and exclusion criteria for this study has been published previously (Maharaj *et al.,* 2018). Healthy controls in this study were volunteers selected randomly from the general population including those employed at the hospital, healthy unrelated people accompanying the patient with no history of any inflammatory arthritis, or any comorbidities and healthy volunteers recruited through word of mouth.

### *MTHFR* C677T rs1801133 and *TP53* Pro72Arg rs1042522 genotyping

The FlexiGene^®^ DNA isolation kit (Qiagen) was used to isolate genomic DNA from whole blood taken from PsA patients and healthy controls as described in a previously published study (Maharaj *et al*., 2018). Thereafter, the *TP53* Pro72Arg rs1042522 and *MTHFR* C677T rs1801133 genotyping was performed using the polymerase chain reaction-restriction fragment length polymorphism (PCR-RFLP) method. The GoTaq^®^ G2 Flexi DNA Polymerase PCR kit (Promega) and CFX96 Touch^TM^ Real-Time PCR Detection System (Bio-Rad) was used to amplify the 131 bp (*TP53* rs1042522) and 198 bp (*MTHFR* rs1801133) PCR products. The 30µl reaction consisted of 1X Green GoTaq Flexi buffer, 2.5 mM MgCl_2_, 200 µM of each dNTP (A, T, G, C), 0.2 Units GoTaq Flexi DNA polymerase, 20 pmol (*TP53* rs1042522) or 40 pmol (*MTHFR* rs1801133) of each forward and reverse primers (Inqaba Biotec) and 30 ng genomic DNA template. The forward and reverse primer sequences were as follows: (i) *TP53* rs1042522 (forward: 5′-TTGCCGTCCCAAGCAATGGATGA-3′ and reverse: 5′-TCTGGGAAGGGACAGAAGATGAC-3′) (Khan *et al.,* 2016), and (ii) *MTHFR* rs1801133 (forward: 5′-TGAAGGAGAAGGTGTCTGGGGGA-3′ and reverse: 5′-AGGACGGTGCGGTGAGAGTG-3′) (Ramkaran *et al.,* 2015). A no-template DNA sample was used to rule out PCR contamination at the DNA level. The PCR conditions were as follows: 

(i) *TP53* rs1042522: 96°C for 12 min (initial denaturation), followed by 35 cycles at 94°C for 30 sec (denaturation), 55°C for 30 sec (annealing) and 72°C for 30 sec (extension). This was followed by a final extension at 72°C for 5 min (Khan *et al.,* 2016). 

(ii) *MTHFR* rs1801133: 95°C for 5 min (initial denaturation), followed by 35 cycles at 95°C for 1 min (denaturation), 59°C for 1 min (annealing) and 72°C for 2 min (extension). This was followed by a final extension at 72°C for 7 min (Ramkaran *et al.,* 2015).

Thereafter, the PCR products and DNA ladder were electrophoresed on 1.8% agarose gel containing 2µl GelRed and visualised using the ChemiDoc™ XRS+ Molecular Imaging System (Bio-Rad) in order to verify the amplification, integrity and size of the 131 bp (*TP53* rs1042522) and 198 bp (*MTHFR* rs1801133) PCR products. The PCR products were then subjected to an overnight digestion for 14 hrs at 60°C (*TP53* rs1042522) and 14 hrs at 37°C (*MTHFR* rs1801133) by using the *BstU* I and *Hinf* I restriction enzymes (New England BioLabs), respectively. The restricted PCR products and DNA ladder were electrophoresed on 3% agarose gels containing 2µl GelRed and visualised as mentioned above for SNP detection in PsA patients and healthy controls. SNP identification were as follows:

(i) *TP53* rs1042522: The homozygous wild-type genotype (Pro/Pro genotype; no cleavage of the 131 bp PCR product). The heterozygous variant genotype (Pro/Arg genotype; yielded 3 bands of 131 bp, 81 bp and 50 bp). The homozygous variant genotype (Arg/Arg genotype; yielded 2 bands of 81 bp and 50 bp) (Khan *et al.,* 2016).

(ii) *MTHFR* rs1801133: The homozygous wild-type genotype (CC genotype; no cleavage of the 198 bp PCR product). The heterozygous variant genotype (CT genotype; yielded 3 bands of 198 bp, 175 bp and 23 bp). The homozygous variant genotype (Arg/Arg genotype; yielded 2 bands of 175 bp and 23 bp) (Ramkaran *et al.,* 2015).

### Statistical analysis

**Figure 1 -:**
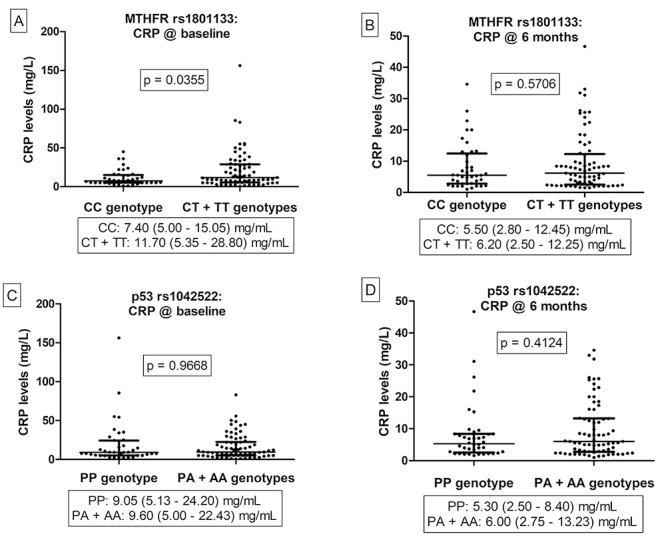
Blood CRP levels in PsA patients genotyped for the *MTHFR* rs1801133 (A and B) and *TP53* rs1042522 (C and D) gene variants. CRP levels were detected when patients were initially recruited into the study (at baseline) and after undergoing 6 months of methotrexate treatment.

STATA version 17.0 statistical software package was used for data analysis. Data were analysed using the Mann-Whitney U test (Figure 1), multivariate regression analysis (Table S1 and Table S2), and Chi-squared (χ2) test and Fisher’s exact test (Table 1, Table 2 and Table 3). Data in Figure 1 were assessed for normality using the Shapiro-Wilk test and are presented as the median and interquartile range (IQR), multivariate regression analysis data are represented as the beta coefficient (β), and Fisher’s exact test data are represented as the odds ratio (OR) and relative risk ratio (RR) at 95% confidence intervals (CI). A *p* value less than 0.05 was considered statistically significant. 

### Ethics approval and consent to participate

The study received ethical approval from the Pharma-Ethics Research Ethics Committee of South Africa (Reference number: 13095660). All participants signed informed consent forms prior to enrolment.

### Informed consent

The study was approved by the Institutional Ethics Committee and was carried out in accordance with The Code of Ethics of the World Medical Association for experiments involving humans.

## Results

### Demographical, clinical and biochemical characteristics of PsA patients

Table 1 highlights the demographics and clinical characteristics of PsA patients. PsA was most prevalent amongst the 41-60 years age groups and in Indians (74%), majority were overweight and obese (74%), 19% were active smokers and there was a similar prevalence between males (54%) and females (46%). Majority tested negative for RF-IgM (96%), underwent radiology (71%) and were on methotrexate medication (95%). Patients had a median HAQ score [0.38 (0.0 - 1.0)] indicative of minimal functional impairments from the PsA (Table 1). Significance was noted for median CRP levels taken at baseline [9.15 (5 - 23.63) mg/L] and after 6 months of treatment [5.70 (2.75 - 12.13) mg/L] (p = 0.0001) (data not displayed in Table 1). Majority had high CRP (> 3 mg/L; baseline: 86% and after 6 months: 71%) and LDL cholesterol (> 3 mmol/L; 61%) levels, and displayed 25(OH)D level indicative of deficiency (≤ 20 ng/ml; 60%) and insufficiency (20.1 - 29.9 ng/ml; 25%). Around 51% and 54% of patients had high total cholesterol and HDL cholesterol levels, respectively. Based on the fasting blood glucose levels, impaired glucose tolerance and prediabetes (plasma glucose: 5.6 - 6.9 mmol/L), diabetes (plasma glucose: > 7 mmol/L) and high HbA1c levels (> 6%) was prevalent amongst 26%, 21% and 29% of patients, respectively (Table 1). 

**Table 1 -:** Demographics and clinical characteristics of patients with PsA (n = 114) and healthy controls (n = 100).

Parameters	PsA patients (n = 114)	Healthy controls (n = 100)	p-Value
Age (years), n (%): 18 - 30 31 - 40 41 - 50 51 - 60 61 - 77	8 (7) 12 (11) 38 (33) 31 (27) 25 (22)	15 (15) 20 (20) 32 (32) 17 (17) 16 (16)	0.0434
Sex, n (%): Male Female	62 (54) 52 (46)	35 (35) 65 (65)	0.0058
Race, n (%): Indian Caucasian	84 (74) 30 (26)	62 (62) 38 (38)	0.0781
Body mass index (BMI) (kg/m^2^), n (%): BMI < 25.0 BMI > 25.0	30 (26) 84 (74)	27 (27) 73 (73)	1.000
Smoking status, n (%): Yes No	22 (19) 92 (81)		
Radiology, n (%): Yes No	81 (71) 33 (29)		
Health Assessment Questionnaire (HAQ) score, n (%): < 0.5 > 0.5	59 (52) 55 (48)		
Rheumatoid factor-immunoglobulin M (RF-IgM), n (%): Positive Negative	5 (4) 109 (96)		
Medications, n (%): Methotrexate Sulfasalazine Leflunomide Biologics ^*^	108 (95) 31 (27) 21 (18) 9 (8)		
C-reactive protein (CRP) (mg/L), n (%): @ Inclusion: ≤ 1.0 1.1 - 3 > 3 @ 6 months ≤ 1.0 1.1 - 3 > 3	3 (3) 13 (11) 98 (86) 1 (1) 32 (28) 81 (71)		
Fasting plasma glucose (mmol/L), n (%): ≤ 5.5 5.6 - 6.9 ≥ 7.0	60 (53) 30 (26) 24 (21)		
Total cholesterol (mmol/L), n (%): ≤ 5 > 5	58 (51) 56 (49)		
Low density lipoprotein (LDL) cholesterol (mmol/L), n (%): ≤ 3 > 3	44 (39) 70 (61)		
High density lipoprotein (HDL) cholesterol (mmol/L), n (%): > 1.1 < 1.1	62 (54) 52 (46)		
Blood glycated haemoglobin (HbA1c) (%), n (%): ≤ 6 > 6	81 (71) 33 (29)		
25-hydroxy vitamin D [25(OH)D] (ng/ml), n (%): ≤ 20 20.1 - 29.9 ≥ 30	68 (60) 29 (25) 17 (15)		

^*^ Biologics: etanercept, adalimumab and infliximab. PsA: psoriatic arthritis. The *p* values generated for Age was calculated using the Chi-squared test. The *p* values generated for Sex, Race and BMI were calculated using the Fisher’s exact test. A *p* < 0.05 was considered as being significant. BMI: (normal: < 25.0 kg/m^2^, overweight and obese: > 25.0 kg/m^2^). HAQ score: (minimal functional impairments from the PsA: < 0.5, moderate to severe functional impairments from the PsA: > 0.5). CRP: (low: ≤ 1.0 mg/L, moderate: 1.1 - 2 mg/L, high: > 3 mg/L). Fasting plasma glucose: (normal: ≤ 5.5 mmol/L, impaired glucose tolerance/ pre-diabetes: 5.6 - 6.9 mmol/L, diabetes: ≥ 7.0). Total cholesterol: (low: ≤ 5 mmol/L, high: > 5 mmol/L). LDL cholesterol: (low: ≤ 3 mmol/L, high: > 3 mmol/L). HDL cholesterol: (low: ≤ 1.1 mmol/L, high: > 1.1 mmol/L). HbA1c: (low: ≤ 6 %, high: > 6 %). 25(OH)D: (deficiency: ≤ 20 ng/ml, insufficiency: 20.1 - 29.9 ng/ml, sufficiency: > 30 ng/ml).

Data for multivariate regression analysis associating race, sex, BMI and smoking status with clinical and biochemical parameters in patients with PsA are presented in Table S1. (i) Race: In comparison to Caucasians, Indian PsA patients had higher baseline CRP levels (unadjusted β = 9.18, p = 0.041) and lower HDL (unadjusted β = -0.14, p = 0.024 and adjusted β = -0.16, p = 0.012) and 25(OH)D (unadjusted β = -7.48, p = 0.002 and adjusted β = -7.98, p = 0.001) levels. (ii) Sex: In comparison to males, female PsA patients had reduced disease duration (adjusted β = -2.75, p = 0.031) and plasma glucose levels (adjusted β = -0.83, p = 0.052) and higher HDL levels (unadjusted β = 0.15, p = 0.006 and adjusted β = 0.13, p = 0.018). (iii) BMI: In comparison to the normal BMI group (< 25.0 kg/m^2^), overweight and obese patients (> 25.0 kg/m^2^) had higher LDL (adjusted β = 0.63, p = 0.027) and lower HDL (unadjusted β = -0.17, p = 0.006 and adjusted β = -0.13, p = 0.088) levels. (iv) Smoking status: In comparison to non-smokers, patients who were active smokers had high total cholesterol (unadjusted β = 0.52, p = 0.038 and adjusted β = 0.61, p = 0.019) and LDL cholesterol (unadjusted β = 0.54, p = 0.028 and adjusted β = 0.56, p = 0.028) levels. (Table S1).

### Genotype and allele frequencies for PsA patients and healthy controls genotyped for the *MTHFR* rs1801133 and *TP53* rs1042522

Table 2 and Table 3 highlights the genotypes and allele frequencies for PsA patients and healthy controls genotyped for the *MTHFR* C677T rs1801133 and *TP53* Pro72Arg rs1042522 and further stratified based on race, respectively. (i) *MTHFR* rs1801133: There was a marginally significant genotype distribution between all PsA patients and healthy controls (CC, CT, TT: 36%, 57%, and 7% versus 49%, 49%, and 2%; p = 0.0589), and PsA patients had a significantly higher frequency of the variant T-allele versus healthy controls (36% versus 26.5%; OR = 1.53, 95% CI = 1.01 - 2.31, p = 0.0477). In addition, Caucasian PsA patients had a significantly higher frequency of the variant T-allele versus healthy Caucasian controls (40% versus 22%; OR = 2.31, 95% CI = 1.10 - 4.88, p = 0.0379). No significant association was observed between Indian PsA patients and Indian healthy controls (Table 2). (ii) *TP53* rs1042522: PsA patients had a significantly higher frequency of the variant Arg-allele versus healthy controls (41% versus 31%; OR = 1.56, 95% CI = 1.05 - 2.33, p = 0.0344). In addition, Indian PsA patients had a significantly higher frequency of the variant Arg-allele versus healthy Indian controls (42% versus 29%; OR = 1.75, 95% CI = 1.07 - 2.86, p = 0.0275). No significant association was observed between Caucasian PsA patients and Caucasian healthy controls (Table 3).

**Table 2 -:** Genotype and allele frequencies for PsA patients and healthy controls genotyped for the *MTHFR* rs1801133 and stratified based on race.

Frequency, n (%)	Healthy controls	PsA patients	*p* Value	RR (95% CI)	OR (95% CI)
All PsA patients (n = 114) and healthy controls (n = 100)					
Genotype, n (%)					
CC	49 (49)	41 (36)	0.0589^a1^		
CT	49 (49)	65 (57)			
TT	2 (2)	8 (7)			
CT+TT	51 (51)	73 (64)	0.0710^b^	1.32 (1.0 - 1.76)	1.71 (0.99 - 2.96)
Allele, n (%)					
C	147 (73.5)	147 (64)	0.0477^c^	1.26 (1.0 - 1.61)	1.53 (1.01 - 2.31)
T	53 (26.5)	81 (36)			
Indian PsA patients (n = 84) and Indian healthy controls (n = 62)					
Genotype, n (%)					
CC	26 (42)	29 (35)	0.3422^a2^		
CT	36 (58)	53 (63)			
TT	0 (0)	2 (2)			
CT+TT	36 (58)	55 (65)	0.3911^b^	1.20 (0.82 - 1.74)	1.37 (0.70 - 2.69)
Allele, n (%)					
C	88 (71)	111 (66)	0.4459^c^	1.14 (0.85 - 1.54)	1.26 (0.76 - 2.08)
T	36 (29)	57 (34)			
Caucasian PsA patients (n = 30) and Caucasian healthy controls (n = 38)					
Genotype, n (%)					
CC	23 (61)	12 (40)	0.0993^a3^		
CT	13 (34)	12 (40)			
TT	2 (5)	6 (20)			
CT+TT	15 (39)	18 (60)	0.1423^b^	1.45 (0.93 - 2.25)	2.30 (0.86 - 6.12)
Allele, n (%)					
C	59 (78)	36 (60)	0.0379^c^	1.50 (1.01 - 2.23)	2.31 (1.10 - 4.88)
T	17 (22)	24 (40)			

^a^ ^1, a2, a3^Chi squared p value (controls genotypes vs. PsA patients’ genotypes) (^a1^ χ^2^ = 5.665, 2 DF; ^a2^ χ^2^ = 2.144, 2 DF; ^a3^ χ^2^ = 4.620, 2 DF). ^b^Fisher’s exact test p value (CC vs. CT+TT genotypes). ^c^Fisher’s exact test p value (controls C and T alleles vs. PsA patients C and T alleles). A *p* < 0.05 was considered as being significant. C: cytosine; CI: confidence interval; DF: degrees of freedom; RR: relative risk; OR: odds ratio; PsA: psoriatic arthritis; T: thymine; χ^2^: Chi-squared test.

**Table 3 -:** Genotype and allele frequencies for PsA patients and healthy controls genotyped for the *TP53* rs1042522 and stratified based on race.

Frequency, n (%)	Healthy controls	PsA patients	*p* Value	RR (95% CI)	OR (95% CI)
All PsA patients (n = 114) and healthy controls (n = 100)					
Genotype, n (%)					
PP	48 (48)	40 (35)	0.0971^a1^		
PA	42 (42)	54 (47)			
AA	10 (10)	20 (18)			
PA+AA	52 (52)	74 (65)	0.0702^b^	1.32 (1.0 - 1.75)	1.71 (0.99 - 2.96)
Allele, n (%)				
P	138 (69)	134 (59)	0.0344^c^	1.28 (1.02 - 1.60)	1.56 (1.05 - 2.33)
A	62 (31)	94 (41)			
Indian PsA patients (n = 84) and Indian healthy controls (n = 62)					
Genotype, n (%)					
PP	31 (50)	31 (37)	0.0839^a2^		
PA	26 (42)	36 (43)			
AA	5 (8)	17 (20)			
PA+AA	31 (50)	53 (63)	0.1294^b^	1.36 (0.93 - 1.97)	1.71 (0.88 - 3.33)
Allele, n (%)					
P	88 (71)	98 (58)	0.0275^c^	1.39 (1.03 - 1.89)	1.75 (1.07 - 2.86)
A	36 (29)	70 (42)			
Caucasian PsA patients (n = 30) and Caucasian healthy controls (n = 38)					
Genotype, n (%)					
PP	17 (45)	9 (30)	0.3382^a3^		
PA	16 (42)	18 (60)			
AA	5 (13)	3 (10)			
PA+AA	21 (55)	21 (70)	0.3150^b^	1.31 (0.87 - 1.97)	1.89 (0.69 - 5.18)
Allele, n (%)					
P	50 (66)	36 (60)	0.5914^c^	1.12 (0.81 - 1.54)	1.28 (0.64 - 2.59)
A	26 (34)	24 (40)			

^a^ ^1, a2, a3^Chi squared p value (controls genotypes vs. PsA patients’ genotypes) (^a1^ χ^2^ = 4.665, 2 DF; ^a2^ χ^2^ = 4.956, 2 DF; ^a3^ χ^2^ = 2.168, 2 DF). ^b^Fisher’s exact test p value (PP vs. PA+AA genotypes). ^c^Fisher’s exact test p value (controls P and A alleles vs. PsA patients P and A alleles). A *p* < 0.05 was considered as being significant. A: Arginine; CI: confidence interval; DF: degrees of freedom; OR: odds ratio; P: proline; PsA: psoriatic arthritis; RR: relative risk; χ^2^: Chi-squared test.

### Association between *MTHFR* rs1801133 and *TP53* rs1042522 variants and clinical and biochemical parameters in PsA patients

*MTHFR rs1801133*: PsA patients with the variant CT + TT genotypes had significantly higher median CRP levels at baseline (on inclusion) versus those with the wildtype CC genotypes (11.70 (5.35 - 28.80) mg/mL versus 7.40 (5.00 - 15.05) mg/mL, p = 0.0355) (unadjusted β = 9.80, p = 0.018 and adjusted β = 9.24, p = 0.027). However, after 6 months of methotrexate treatment median CRP levels between genotypes reduced and remained similar (p=0.5706) ( Table S2 and Figure 1). (ii) *TP53* rs1042522: No association was observed (Table S2 and Figure 1). 

## Discussion

Several modifiable risk factors play a role in the pathogenesis of PsA, including being overweight and obesity (each kg/m^2^ rise in BMI was associated with approximately 6% increase in PsA prevalence) (Xie *et al.,* 2021), cigarette smoking (nicotine binds to the nicotinic acetylcholine receptors in various cell types such as thymocytes, leukaemic cells, B-cells and T-cells, leading to aberrant functionality of keratinocytes and psoriasis and PsA progression) (Højgaard *et al.,* 2015, Maharaj *et al.,* 2018), aberrantly high cholesterol levels (Wu *et al.,* 2014), impaired glucose tolerance and diabetes mellitus (Eder *et al.,* 2014), and 25(OH)D deficiency (Filoni *et al.,* 2018). All the aforementioned play a contributing role towards the manifestation of metabolic syndrome which is highly prevalent in patients with PsA (Raychaudhuri *et al.,* 2010). Non-modifiable risk factors, including age (Queiro *et al.,* 2013 a ), sex (Queiro *et al.,* 2013b), race (Leung *et al.,* 2017), and genetic variants (SNP) (Liu *et al.,* 2008) also play a role in the pathogenesis of PsA. Patients with uncontrolled PsA tend to have abnormally high levels of CRP, a biomarker of inflammation and disease severity, which could be reversed upon treatment with DMARDs (Asahina *et al.,* 2016). Similar findings were reported in the present study (Table 1 and Table S1).

Higher baseline levels of CRP were observed in Indian PsA patients in this study which might indicate a more pronounced inflammatory response compared to patients from other populations. This could be influenced by genetic, environmental and lifestyle, or other health-related factors specific to the Indian population (Azmi and Victora, 2007). Female PsA patients in this study had reduced disease duration. This could be due to the fact that: (i) women might seek medical attention sooner than men due to more pronounced symptoms or higher health awareness, leading to an earlier diagnosis, (ii) PsA could manifest differently in men and women, potentially influencing the perceived duration and progression of the disease, (iii) biological differences, including genetic and hormonal influences, might affect the progression and duration of PsA in women, and (iv) women might have different patterns of healthcare utilization, which can impact the timing of diagnosis and treatment (Westergaard *et al*., 2019).

Limited and conflicting data are available as to whether the *MTHFR* rs1801133 and *TP53* rs1042522 play a role in psoriasis and PsA development in different ethnic race groups. The *MTHFR* rs1801133 was reported to be associated with the pathogenesis of psoriasis and PsA in Chinese (Baiqiu *et al.,* 2000) and European Caucasian (Vasku *et al.,* 2009) patients. Conversely, no association was found in Austrian (Weger *et al.,* 2008) and Malaysian (Liew *et al.,* 2012) patients. An association between the *TP53* rs1042522 and psoriasis and PsA pathogenesis was observed in a cohort of European Caucasians (Assmann *et al.,* 2010) but not in Canadian Caucasians (Butt *et al.,* 2006). Beckman *et al*. (1994) found a strong correlation between the *TP53* rs1042522 and ethnicity. The frequency of the variant Arg-allele was found to be most predominant in populations living farther away from the equator (similar to our study population in South Africa), and it was concluded that the *TP53* rs1042522 was balanced and maintained by natural selection (Beckman *et al.,* 1994). 

In this study, the *MTHFR* rs1801133 and *TP53* rs1042522 was associated with PsA (Caucasians and Indians combined) and was more specifically associated with PsA development in South African Caucasians and Indians, respectively (Table 2 and Table 3). A significantly higher frequency of the variant *TP53* Arg-allele (rs1042522) was observed in PsA patients versus healthy controls, and Indian PsA patients had a significantly higher frequency of the *TP53* variant Arg-allele versus healthy Indian controls. The p53 protein plays a role in regulating the immune response and inflammation. Variants of the *TP53* gene, such as the Arg allele, might alter the inflammatory pathways, potentially leading to an increased susceptibility to inflammatory diseases like PsA. The *TP53* Arg allele might influence the mechanism of PsA by affecting the regulation of cell growth, apoptosis, and the immune response. These changes can contribute to the chronic inflammation and joint damage seen in PsA patients. A higher frequency of the *TP53* Arg allele in PsA patients compared to healthy controls suggests a genetic predisposition. Individuals carrying this variant might be more prone to developing PsA due to the altered regulatory functions of the p53 protein (Salvador *et al*., 2005). There was also a lack of significant association between Indian PsA patients and health controls for the *MTHFR* rs1801133 variant, and Caucasian PsA patients and health controls for the *TP53* rs1042522 variant suggesting that this specific genetic variant does not play a major role in the susceptibility to PsA within these population. The impact of a SNP on disease can differ between populations due to several factors, including genetic background, gene-environment interactions, environmental influences, population-specific adaptations, historical selection pressures, adaptive evolution, linkage disequilibrium and epigenetic factors (Hettiarachchi and Komar, 2022).

As mentioned, *MTHFR* is involved in the metabolism of homocysteine and folic acid (Engbersen *et al.,* 1995, Liew and Gupta, 2015). The *MTHFR* rs1801133 is associated with elevated plasma homocysteine levels (Jacques *et al.,* 1996, Liew *et al.,* 2012, Liew and Gupta, 2015). Elevated homocysteine levels (Catena *et al.,* 2014), and the *MTHFR* rs1801133 (van Winkel *et al.,* 2010) are risk factors associated with the development of metabolic syndrome. Hyperhomocysteinaemia is also associated with folate and vitamin B12 deficiency (Muskiet, 2005, Ramkaran *et al.,* 2015). Malerba *et al*. (2006) reported that patients with chronic plaque psoriasis had exacerbated plasma homocysteine levels and lower plasma folic acid levels (Malerba *et al.,* 2006). Similar findings were found in patients with PsA (Kural *et al.,* 2003). The combination of aberrantly elevated plasma CRP and homocysteine levels alone were considered to be useful prognostic biomarkers in predicting the severity of cardiovascular disorders in comparison to monitoring CRP and homocysteine levels independently (Yan *et al.,* 2010).

PsA is exceedingly rare in the indigenous African South African population (Maharaj and Tak, 2015). Nienaber-Rousseau *et al*. (2013) genotyped Black South Africans for the *MTHFR* rs1801133 and correlated these findings with plasma homocysteine levels. Around 84%, 16% and 0.8% of the study population had the homozygous wild-type CC, heterozygous variant CT and homozygous variant TT genotypes, respectively. Individuals with the CC and TT genotypes had the lowest and highest homocysteine levels, respectively. It was proposed that Black South Africans were at a lesser risk of developing hyperhomocysteinaemia-related disorders such as psoriasis and PsA due to the low prevalence of the *MTHFR* rs1801133 (Nienaber-Rousseau *et al.,* 2013). This could be a reason behind the high prevalence of PsA in South African Caucasians and Indians with the *MTHFR* rs1801133 variant CT+TT genotypes (Table 2 and Table 3). 

Methotrexate (a folic acid antagonist) is commonly used to treat moderate to severe PsA; it mechanistically inhibits the dihydrofolate reductase (DHFR) enzyme. As a result, the conversion of dihydrofolate (DHF) into tetrahydrofolate (THF) is prevented (Auepemkiate, 2016). Methotrexate also has other effects; it negatively affects protein and DNA synthesis and interferes with the growth and proliferation of both normal and abnormal cells. As a consequence, this can trigger the activation of both pro-inflammatory and anti-inflammatory cytokines, leading to inflammation and elevated CRP levels (van Ede *et al.,* 1998, Haustein and Rytter*,* 2000, Auepemkiate, 2016,). In the present study, over 95% of PsA patients were on methotrexate medication which, in combination with other DMARDs, significantly reduced CRP levels after 6 months of treatment compared to high CRP levels recorded at baseline (Table 1). In addition, baseline CRP levels in PSA patients with the *MTHFR* rs1801133 variant CT+TT genotypes were significantly higher compared to those with the homozygous wild-type CC genotype. However, treatment with methotrexate, in combination with other DMARDs, reduced and normalized CRP levels between genotypes and significance was not noted (Figure 1 and Table S2). Findings suggest that PsA patients with the *MTHFR* rs1801133 variant T-allele are more susceptible to having higher CRP levels in comparison to patients with the wild-type C-allele which could be reversed by methotrexate therapy. 

## Conclusion

In conclusion, age (> 40 years), sex, race, cigarette smoking, obesity, elevated CRP and LDL-cholesterol levels, and 25(OH)D deficiency are some of the risk factors associated with the pathogenesis of PsA within the context of a South African population consisting of Caucasians and Indians. The *MTHFR* rs1801133 (predominant in Caucasians), and to a lesser extent *TP53* rs1042522 (predominant in Indians), may play a role in the pathogenesis of PsA. In addition, PsA patients with the *MTHFR* rs1801133 variant CT+TT genotypes are more at risk in having higher CRP levels which could be ameliorated by methotrexate therapy. Study limitation includes sample size and further studies are warranted in a bigger cohort to offer more clarity, and to profile the expression of *MTHFR* and *TP53* in the case-control cohorts. 

## Supplementary material

The following online material is available for this article:

Table S1 - Multivariate regression analysis associating race, sex, BMI and smoking status with clinical and biochemical parameters in patients with PsA.

Table S2 - Multivariate regression analysis associating *TP53* rs1042522 and *MTHFR* rs1801133 with clinical and biochemical parameters in patients with PsA.
