# Supplementary material for: MTHFR C677T rs1801133 and TP53 Pro72Arg rs1042522 gene variants in South African Indian and Caucasian psoriatic arthritis patients
Source: Genet Mol Biol. 2025 Jan 10;48(1):e20230325. doi: 10.1590/1678-4685-GMB-2023-0325 (PMC11721215; doi:10.1590/1678-4685-GMB-2023-0325)
Supplement: Table S1 - [file 1415-4757-GMB-48-1-e20230325-s1.pdf]

*Genetics and Molecular Biology*
https://doi.org/10.1590/1678-4685-GMB-2023-0325

# Supplementary Material to "*MTHFR* C677T rs1801133 and *TP53* Pro72Arg rs1042522 gene variants in South African Indian and Caucasian psoriatic arthritis patients"

**Table S1 -** Multivariate regression analysis associating race, sex, BMI and smoking status with clinical and biochemical parameters in patients with PsA.

| **Parameter** | **Model (M)** | **Race** | | **Sex** | | **BMI** | | **Smoking** | |
|---|---|---|---|---|---|---|---|---|---|
| | | **β** | **p** | **β** | **p** | **β** | **p** | **β** | **p** |
| | | Reference: Caucasians | | Reference: Males | | Reference: BMI < 25.0 kg/m$^2$ | | Reference: No | |
| HAQ score | M1 | 0.08 | 0.577 | 0.22 | 0.074 | -0.07 | 0.627 | -0.08 | 0.593 |
| | M2 | 0.15 | 0.294 | 0.20 | 0.132 | -0.22 | 0.232 | -0.00 | 0.976 |
| Disease duration (years) | M1 | -2.41 | 0.092 | -1.67 | 0.189 | -0.41 | 0.775 | -1.92 | 0.231 |
| | M2 | -1.96 | 0.175 | -2.75 | 0.037 | -1.47 | 0.428 | -2.40 | 0.139 |
| CRP on inclusion (mg/L) | M1 | 9.18 | 0.041 | 0.30 | 0.939 | -7.30 | 0.104 | 2.68 | 0.595 |
| | M2 | 7.04 | 0.128 | 2.24 | 0.589 | -6.11 | 0.300 | 2.45 | 0.635 |
| CRP @ 6 months (mg/L) | M1 | 2.44 | 0.195 | 1.50 | 0.371 | -0.98 | 0.606 | 0.87 | 0.678 |
| | M2 | 2.37 | 0.228 | 2.19 | 0.217 | -1.97 | 0.433 | 1.25 | 0.569 |
| Plasma glucose (mmol/L) | M1 | -0.26 | 0.574 | -0.58 | 0.153 | 0.56 | 0.219 | -0.61 | 0.233 |
| | M2 | -0.09 | 0.850 | -0.83 | 0.052 | 0.25 | 0.675 | -0.85 | 0.107 |
| Total cholesterol (mmol/L) | M1 | -0.10 | 0.655 | 0.16 | 0.408 | 0.21 | 0.354 | 0.52 | 0.038 |
| | M2 | -0.15 | 0.504 | 0.31 | 0.136 | 0.47 | 0.112 | 0.61 | 0.019 |

| | | | | | | | | | |
|---|---|---|---|---|---|---|---|---|---|
| LDL cholesterol (mmol/L) | M1 | 0.00 | 0.984 | 0.02 | 0.912 | 0.37 | 0.095 | 0.54 | 0.028 |
| | M2 | -0.08 | 0.734 | 0.20 | 0.317 | 0.63 | 0.027 | 0.56 | 0.028 |
| HDL cholesterol (mmol/L) | M1 | -0.14 | 0.024 | 0.15 | 0.006 | -0.17 | 0.006 | -0.09 | 0.201 |
| | M2 | -0.16 | 0.012 | 0.13 | 0.018 | -0.13 | 0.088 | -0.03 | 0.633 |
| HbA1c (%) | M1 | 0.08 | 0.767 | -0.30 | 0.206 | 0.29 | 0.272 | -0.14 | 0.643 |
| | M2 | 0.24 | 0.383 | -0.39 | 0.116 | -0.18 | 0.596 | -0.26 | 0.392 |
| 25(OH)D (ng/ml) | M1 | -7.48 | 0.002 | 0.73 | 0.733 | -0.40 | 0.867 | -4.72 | 0.079 |
| | M2 | -7.98 | 0.001 | -0.76 | 0.725 | 3.16 | 0.301 | -4.39 | 0.103 |

M1: Unadjusted model. M2: Model adjusted for age, sex, race, BMI and smoking status. BMI: (normal: < 25.0 kg/$m^2$, overweight and obese: > 25.0 kg/$m^2$). 25(OH)D: 25-hydroxy vitamin D; β: Beta coefficient; BMI: Body mass index; CRP: C-reactive protein; HAQ: Health assessment questionnaire; HbA1c: Blood glycated haemoglobin; HDL: High density lipoprotein; LDL: Low density lipoprotein; M: Model; PsA: Psoriatic arthritis. A $p < 0.05$ was considered as being significant.
