# Supplementary material for: MTHFR C677T rs1801133 and TP53 Pro72Arg rs1042522 gene variants in South African Indian and Caucasian psoriatic arthritis patients
Source: Genet Mol Biol. 2025 Jan 10;48(1):e20230325. doi: 10.1590/1678-4685-GMB-2023-0325 (PMC11721215; doi:10.1590/1678-4685-GMB-2023-0325)
Supplement: Table S2 - [file 1415-4757-GMB-48-1-e20230325-s2.pdf]

Genetics and Molecular Biology
https://doi.org/10.1590/1678-4685-GMB-2023-0325

Supplementary Material to "*MTHFR* C677T rs1801133 and *TP53* Pro72Arg rs1042522 gene variants in South African Indian and Caucasian psoriatic arthritis patients"

Table S2 - Multivariate regression analysis associating *TP53* rs1042522 and *MTHFR* rs1801133 with clinical and biochemical parameters in patients with PsA.

Parameters	Model (M)	*TP53* Pro72Arg rs1042522		*MTHFR* C677T rs1801133	
		β (PA+AA)	p	β (CT+TT)	p
		Reference: Wildtype Genotype (PP)		Reference: Wildtype genotype (CC)	
HAQ score	M1	-0.12	0.370	0.00	0.992
	M2	-0.11	0.392	-0.00	0.985
Disease duration (years)	M1	-0.26	0.844	0.51	0.702
	M2	-0.49	0.705	0.63	0.636
CRP on inclusion (mg/L)	M1	-3.36	0.420	9.80	0.018
	M2	-2.87	0.487	9.24	0.027
CRP @ 6 months (mg/L)	M1	1.52	0.387	1.25	0.474
	M2	1.76	0.316	1.11	0.535
Plasma glucose (mmol/L)	M1	0.32	0.444	0.15	0.717
	M2	0.32	0.443	0.10	0.809
Total cholesterol (mmol/L)	M1	-0.15	0.466	0.02	0.939
	M2	-0.16	0.447	0.06	0.769
LDL cholesterol (mmol/L)	M1	-0.02	0.926	-0.04	0.827
	M2	-0.01	0.976	-0.03	0.899

HDL cholesterol (mmol/L)	M1	0.01	0.918	0.03	0.633
	M2	-0.01	0.872	0.07	0.188
HbA1c (%)	M1	-0.37	0.130	0.09	0.715
	M2	-0.35	0.147	0.01	0.958
25(OH)D (ng/ml)	M1	2.35	0.291	-1.20	0.590
	M2	1.69	0.430	0.21	0.924

M1: Unadjusted model. M2: Model adjusted for age, sex, race, BMI and smoking status. 25(OH)D: 25-hydroxy vitamin D; β: Beta coefficient; A/Arg: Arginine; C: Cytosine; CRP: C-reactive protein; HAQ: Health assessment questionnaire; HbA1c: Blood glycated haemoglobin; HDL: High density lipoprotein; LDL: Low density lipoprotein; M: Model; MTHFR: Methylenetetrahydrofolate reductase; p53: Tumour suppressor protein p53; P/Pro: Proline; T: Thymine. A $p < 0.05$ was considered as being significant.
